# Multi-Shell Nano-CarboScavengers for Petroleum Spill Remediation

**DOI:** 10.1038/srep41880

**Published:** 2017-02-03

**Authors:** Enrique A. Daza, Santosh K. Misra, John Scott, Indu Tripathi, Christine Promisel, Brajendra K. Sharma, Jacek Topczewski, Santanu Chaudhuri, Dipanjan Pan

**Affiliations:** 1 Department of Bioengineering, University of Illinois at Urbana Champaign, Urbana, Illinois 61801, USA; 2 Carle Foundation Hospital, Urbana, Illinois, 61801, USA; 3 Illinois Sustainable Technology Center, Prairie Research Institute, University of Illinois at Urbana Champaign, Champaign, Illinois, 61820, USA; 4 Department of Pediatrics, Northwestern University Feinberg School of Medicine, Stanley Manne Children’s Research Institute, Chicago, Illinois 60611, USA; 5 Applied Research Institute, Champaign, Illinois, 61820, USA

## Abstract

Increasingly frequent petroleum contamination in water bodies continues to threaten our ecosystem, which lacks efficient and safe remediation tactics both on macro and nanoscales. Current nanomaterial and dispersant remediation methods neglect to investigate their adverse environmental and biological impact, which can lead to a synergistic chemical imbalance. In response to this rising threat, a highly efficient, environmentally friendly and biocompatible nano-dispersant has been developed comprising a multi-shelled nanoparticle termed ‘Nano-CarboScavengers’ (NCS) with native properties for facile recovery via booms and mesh tools. NCS treated different forms of petroleum oil (raw and distillate form) with considerable efficiency (80% and 91%, respectively) utilizing sequestration and dispersion abilities in tandem with a ~10:1 (oil: NCS; w/w) loading capacity. In extreme contrast with chemical dispersants, the NCS was found to be remarkably benign in *in vitro* and *in vivo* assays. Additionally, the carbonaceous nature of NCS broke down by human myeloperoxidase and horseradish peroxidase enzymes, revealing that incidental biological uptake can enzymatically digest the sugar based core.

Petroleum and all of its derivatives are a necessity in today’s modern world. Although, the processes involved in its extraction, collection, transportation, and distribution are not free of environmental impairment^1^. Oceanic spills in 2014 recorded a total of 706 million gallons of waste oil entering the ocean^2^ with 4,000 tons of that oil lost to the environment^3^. Many conventional large-scale recovery tactics, such as skimmers paired with booms and suction, partially alleviate the issue, but they leave residual ecologically hazardous hydrocarbon layers and other contaminants on the micrometer scale^4^. This residual hydrocarbon layer can impede sunlight from penetrating ocean water by up to 90%, thereby crippling photosynthesis-dependent aquatic ecosystems^5^,^6^. Highly efficient liquid chemical dispersants^7^ lower surface tension of the oil-water interface and thus disperse the petroleum into the water column for natural bioremediation *via* hydrocarbon degrading organisms^8^,^9^,^10^. Unfortunately, these chemicals produce toxic emulsions with the oil during treatment and thereby disrupts the aquatic remediation process^11^,^12^,^13^,^14^.

Nano-enabled technologies can generate cheaper, quicker, and more efficient methods of oil spill remediation^15^. These technologies cover a variety of mechanisms and compositions including gels^16^,^17^,^18^, nanoparticles^19^,^20^,^21^ nanowires^22^,^23^ and magnetic composites^24^,^25^,^26^,^27^ that are commonly centralized around hydrophobicity as a means to attract and capture immiscible hydrocarbons. For example, Zhu *et al*.^28^ recently developed a polyurethane sponge with a hydrophobic polysiloxane coating for high durability and oil absorption capabilities^29^,^30^. Some nano-architectures also use amphiphilic hybrid materials to create complex shelled particles for multipurpose sequestration devices. Wang *et al*.^27^ entrapped negatively charged iron microparticles within micellar arrays and successfully removed hydrophobic organic compounds from soil and media. A more refined crude oil absorbing nano-shelled material used super-hydrophobic magnetic iron oxide nanoparticles cross-linked with a self-assembling amphiphilic polymer for subsequent magnetic recovery post absorption^26^. Even after such successes, oil spill remediation remains a challenge due to the varying composition of raw, unprocessed crude oil and presence of non-petroleum compounds, such as dirt, sulfur, and variable chain length hydrocarbons. Moreover, engineered nanoparticle treatment and recovery from the environment can be overlooked and cause toxic effects to marine life^31^; thus poses a challenge for the makeup of an efficient, non-toxic, and commercially viable product. This environmental susceptability leaves current nanotechnology-based approaches limited to perfecting attributes such as hydrophobicity and porosity. Since nanomaterials can have an extensive impact on petroleum spill remediation^32^, current techniques could benefit from a bio-compatible solid-state dispersive material amenable to large-scale production. Such powdered dispersants can have a profound positive impact on the future of nanomaterial-based remediation and can serve as a safe replacement for toxic chemical dispersants, in addition to having a further absorption function as well. Towards this aim, we have developed a multi-shelled biocompatible nano-agent, termed Nano-CarboScavenger (NCS), with excellent treatment efficiency *via* combined absorption and dispersion properties. To the best of our knowledge, such an approach to creating a powdered dispersant is unique from materials explored thus far.

## Results and Discussion

### Carbon core synthesis and hydrophobic passivation

**Figure 1:**
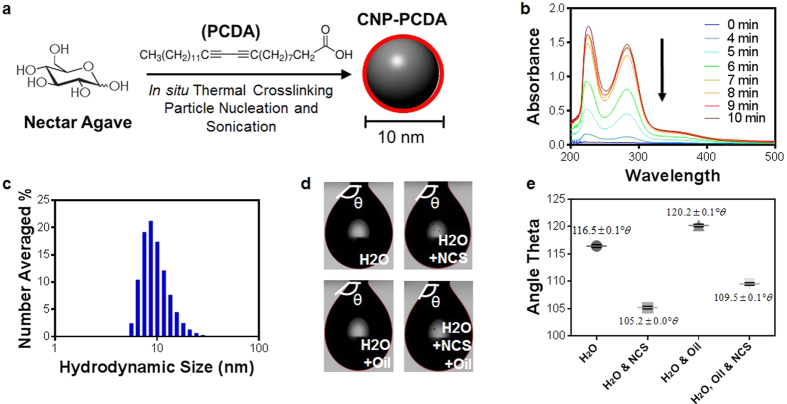
Schematic representation of CNP-PCDA design and synthesis for optimizing dispersion in oil contaminated water. (**a**) Nectar agave is the main carbon source used to produce the carbon core which is subsequently thermally crosslinked with 10,12-pentacosadiynoic acid (PCDA). (**b**) UV-VIS spectrophotometer absorbance measurements at separate time points showing that absorbance increases as PCDA thermally crosslinks with nucleating carbon nanoparticles. (**c**) Number averaged histogram depicting the diameter of CNP-PCDA in THF as measured in THF *via* DLS. (**d**) Goniometer images showing droplet theta angles of water, water exposed to NCS, water exposed to crude oil, water exposed to crude oil then treated with NCS. (**e**) Graph detailing specific theta angle of each sample with error bars in black (n = 30 measurements).

In order to facilitate the dual functionality of a nanomaterial for dispersion/absorption based remediation of diverse petroleum compositions, a multi-component nano-architecture is necessary. Thus we begin with a ‘core first’ approach with inherent dispersive properties of a known biocompatible sugar based carbon nanoparticle^33^,^34^,^35^ (Fig. 1a). As previously mentioned, the ‘dispersion effect’ is caused by lowering the surface tension of the oil-water interface, in order for wave agitation to draw in petroleum droplets with large surface to volume ratios for facile bioremediation *via* bacteria. One of the functional groups found in commercial dispersants responsible for the dispersing activity is the presence of large hydrophobic moieties within the surfactant structure. For this reason, a hydrophobic crosslinker, 10,12-pentacosadiynoic acid (PCDA), is thermally crosslinked onto commercially available agave nectar (an inexpensive sweetener composed of 47–56% of fructose and 19–20% glucose). The formation of the primary hydrophobic layer is a coincidence of the polymerizing PCDA crosslinker^36^,^37^,^38^ to create a stable association with surrounding hydrocarbons through hydrophobic-hydrophobic interactions. As agave nectar and PCDA were hydrothermally crosslinked, samples were extracted and the absorbance of each sample was measured using ultraviolet-visible spectroscopy (UV-VIS) (Fig. 1b). The increase in absorbance peak intensities at wavelength λ_max_ 224 nm and 283 nm indicates *in situ* polymerization of PCDA, and in turn successful crosslinking. Particles were then suspended in tetrahydrofuran (THF) and briefly subjected to probe sonication in an ice bath. The formation of the PCDA-coated carbon nanoparticles (CNP-PCDA) was monitored by dynamic light scattering (DLS) measurements (Fig. 1c). CNP-PCDA had a number averaged hydrodynamic diameter of 8 ± 3 nm (average ± standard deviation) with a polydispersity index (PDI) of 0.39 ± 0.01. Raman scattering of CNP-PCDA revealed the carbonaceous core structure to share graphitic and diamond-like properties with a G/D band ratio of 1.10 (Fig. S1). The attempt to observe the anhydrous state diameter of CNP-PCDA suspended in THF was unsuccessful due to film generation during organic evaporation (Fig. S2).

In order to assess the CNP-PCDA capability to reduce surface tension in an aqueous environment, the CNP-PCDA material was distributed onto the deionized water surface and evaluated *via* goniometer measurements. Judging by the shift in the incident theta angle of water samples under different treatment conditions (water, water exposed to CNP-PCDA, water exposed to crude oil, water exposed to crude oil then treated with CNP-PCDA; Fig. 1d), the CNP-PCDA alone caused a significant reduction in the water’s interfacial surface tension. Therefore, the CNP-PCDA core must be increasing the surface energy for petroleum droplet distribution into the water column for the aforementioned dispersive effects. These results were further confirmed when CNP-PCDA was distributed directly onto crude oil contaminated water and led to highly visible dispersive effects (Video 1).

### Incorporation of absorption properties *via* polymer passivation to generate complete NCS Nano-architecture

**Figure 2:**
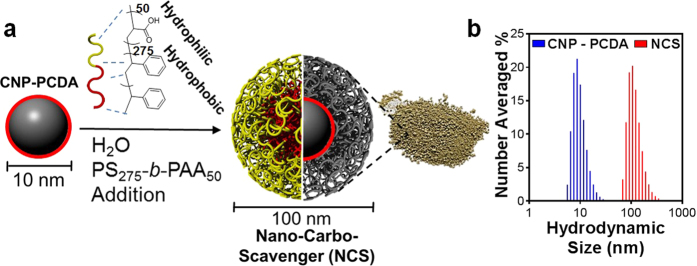
NCS chemistry and synthesis process. (**a**) After CNP-PCDA resuspension, PS-b-PAA is added and self-assembled, entrapping carbon spheres. Powder is shown for visual representation of final NCS product. (**b**) Number averaged histogram depicting the hydrodynamic diameter of NCS in water as measured by DLS. An overlay of the CNP-PCDA histogram is shown for comparison.

As previously studied^20^,^26^,^27^, a polymer based shell approach is able to provide a method for light chained hydrocarbons to adsorb onto the surface of nanoparticles. Thus, to complete the NCS design with an additional absorption feature, improving upon our dispersive core, a uniform self-assembled diblock copolymer serves as an amphiphilic absorption mechanism in addition to biocompatible surface functionalization (Fig. 2a). The self-assembly of amphiphilic polymer polystyrene-block-polyacrylic acid (PS-b-PAA) with a high degree of polymerization (DP) for hydrophobic polystyrene block [i.e. 275] and low DP for hydrophilic polyacrylic acid block [i.e. 50] has been thoroughly studied^39^,^40^,^41^,^42^,^43^,^44^,^45^,^46^,^47^,^48^ and was selected to achieve optimal hydrophobic-hydrophobic interaction with CNP-PCDA core.

In order to verify *in silico* results, raw PS-b-PAA powder was applied to crude oil contaminated water to visualize absorptive effects. The crude oil was seen to absorb onto the polymer particles generating a clump, which is contrary to the dispersive effects seen with the CNP-PCDA core and common surfactants (Video 2). The final stage of NCS synthesis involves the polymeric self-assembly of the outer shell *via* re-suspending CNP-PCDA mass and PS-b-PAA in THF at an optimized 10:2 mass ratio respectively. This is followed by quenching with nanopure water at twice the volume of THF. This solution immediately changed colors from dark brown/red to cloudy, milky beige dictating NCS particle formation.

### Physio-Chemical Properties and Characterization of NCS

DLS measurements revealed that polymer coating increased the hydrodynamic diameter (D_hy_) for NCS to 115 ± 25 nm with a PDI of 0.10 ± 0.01 (Fig. 2b). The D_hy_ of PS-b-PAA particles alone were found to be 75 ± 18 nm with a PDI of 0.16 ± 0.01 (Fig. S3), thus suggesting the encapsulation of multiple CNP-PCDA particles per NCS particle. Assuming the CNP-PCDA randomly pack within the NCS sphere, we can predict the average CNP-PCDA particle per NCS is 30 (packing density coefficientη = 0.64)^49^. The NCS suspension was dehydrated in a vacuum desiccator for four hours and collected as brown powder (Fig. 2a). In order to confirm petroleum remediation ability, 10 mg from each batch of NCS powder was tested on crude oil contaminated water and assessed by visual performance. During batch tests, the NCS were found to immediately disperse the oil upon first contact, followed by slow absorption towards a cohesive clump (Video 3). The powdered sample was preserved in a dark closed container prior to hydrocarbon scavenging applications and physicochemical characterization where it was found to retain its scavenging properties even after 6 months of storage and independent of batch number.

**Figure 3:**
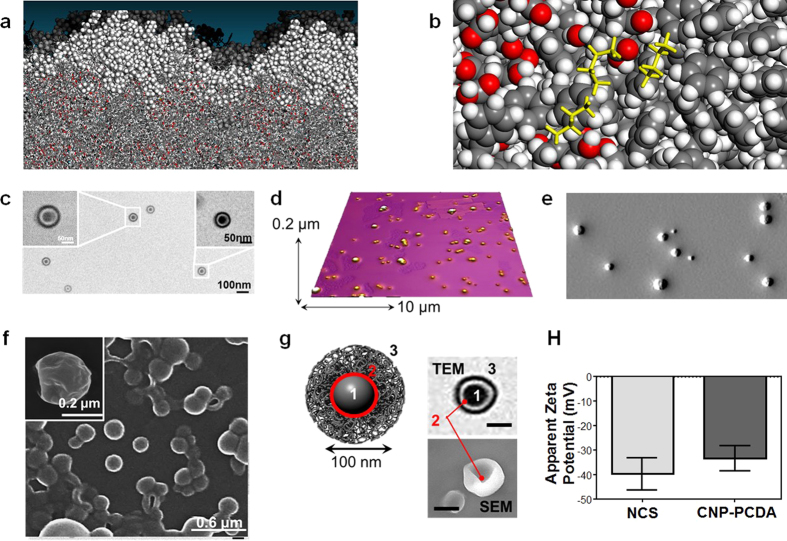
MD Simulation of light chain hydrocarbon interactions and NCS characterization. (**a**) Light distillate oil phase (close-packed spheres) first adsorbs on the PS-b-PAA shell layer (stick models of the polymeric phase) and shows strong adsorption. (**b**) the smaller C_4_H_10_ molecules highlighted in yellow are seen inside the PS-b-PAA layer in more prevalence. (**c**) TEM micrograph of NCS on a carbon-coated grid. Insets show an increased magnification of NCS to visualize shell characteristics. (**d**) Tapping mode AFM surface height heat map of anhydrous NCS on freshly cleaved mica. (**e**) Tapping mode AFM edge deflection image of anhydrous NCS on freshly cleaved mica. Brightness versus darkness indicate ‘increasing’ versus ‘decreasing’ slope in arbitrary units. (**f**) Gold sputtered low voltage (0.5 kV) SEM micrograph of anhydrous NCS on silicon. Inset shows highly detailed surface morphology at a higher magnification. Lower magnification SEM with larger populations of NCS can be found in Fig. S4. (**g**) Theoretical shell structure predicted from TEM (top) and SEM (bottom) insets. Carbon core is labeled as 1, PCDA surface passivation is labeled as 2, and the polymer coating is labeled as 3. (**h**) Zeta potential measurement histogram overlay of NCS and CNP-PCDA in water with a count rate of 285 K counts per second at 12 runs per measurement repeated in triplicates to produce an error bar of one standard deviation.

Although the NCS scavenging ability can be explained through physiochemical explanations, an in silico analysis of the NCS surface interactions with hydrocarbons could provide more detail. The interfacial affinity for different types of hydrocarbons interacting with the self-assembled polymer surface is an important design consideration for improving the performance of NCS as a nano-platform. Molecular dynamics (MD) simulation techniques can be used to identify the different forces that are responsible for optimum performance of the NCS nano-platform. In an ideal case, a superhydrophobic and super-oleophilic shell is needed for NCS to disperse into the oil phase selectively. The breakdown of oil film *via* formation of colloids in water is a function of the adsorption strength of different hydrocarbon molecules on the NCS surface. Subsequently, the adsorption of oil inside the shell will also alter the interfacial free energy of NCS, and oleophilic behaviors can be modified as a function of the NCS shell architecture. A MD simulation-based interface design strategy can be effective in tuning the surface affinity of liquids in polymer nanocomposites^50^. In order to understand the nature of these interactions, a model for the NCS shell layer was constructed using an atomistic model of the polymeric phase. For the NCS particles, the shell of the nanoparticles is simulated using PS-b-PAA. The PS-b-PAA layer composing the outermost shell of the NCS particles is primarily responsible for interaction with petroleum molecules. A simplified distillate oil phase was simulated using hydrocarbons of different chain lengths (C4, C8, C12 hydrocarbons) with roughly 53% of C_4_H_10_, 31% of C_8_H_18_, and 15% of C_12_H_26_ in the mix and with a density of close to 0.8 g/cm^3^. The oil phase was equilibrated using 300 K MD simulation and placed on top of the PS-b-PAA layer for observing the adsorption dynamics. The lateral view of the oil layer adsorbed on the PS-b-PAA shell is shown in Fig. 3a,b. After a prolonged MD simulation at room temperature, the smaller molecules such as C_4_H_10_ are seen to penetrate deep inside the shell layer, and the larger hydrocarbon molecules remained on the surface of the PS-b-PAA shell. This is indicative of the light crude adsorption by the NCS. The shell acted as a mass-selective membrane in letting the smaller molecular fragments inside. For heavier crude, it is conceivable that depending on the molecule size, there is an accumulation of the larger hydrocarbons on the surface. The smaller hydrocarbon molecules inside the PS-b-PAA layer are relatively strongly bound and show saturation effects. In other words, the binding energy and potential energy both increase during simulations as the hydrocarbons enter the shell layers of PS-b-PAA. The NCS can overcome interactions with molecules in the oil film by surface adsorption of the heavy crude, as well as absorption of lower molecular weight components towards the inside of the shell.

Our results indicate that the surface energy interactions between crude oil and NCS are ideal for the selective absorption of small hydrocarbon, and therefore, can reduce the crude oil composition to aid the dispersive ability. Since, heavy crude oil has larger chain hydrocarbons, the surface and surface hydrocarbon binding interaction changes the free energy balance in favor of a breakdown of the oil fill and forming domains where first NCS enters the oil phase, presumably due to low diffusion barrier. On the other hand, it may absorb the large hydrocarbons strongly as shown in MD simulation, and the loss of degrees of freedom of the bound hydrocarbon chain around NCS tilts the free energy balance whereby the nanoparticles floating with the oil lower the generally high surface tension of oil and causes a breakdown of the film, and thus confirms the dual dispersive and absorptive functionality of the proposed NCS Nano-Architecture. The MD simulation results show that heavier molecules get adsorbed on the surface of the shell while the lighter hydrocarbon can penetrate inside the shell. The time frame of MD simulation is limited to observing if the smaller hydrocarbons will eventually reach the core of the NCS. However, even in 500 picoseconds, the molecules have travelled 2–3 nanometers inside the shell. So, it is reasonable to expect that the diffusion of small molecules will continue unless the NCS surface is fully blocked by the larger hydrocarbon molecules. In the dilute case, we fully expect the smaller hydrocarbons to saturate the shell layer in much longer time scales of experiments. Mechanistically, with these simulation results in hand, it seems to be simply a factor of size. The small molecules continue to penetrate the PS-b-PAA shell simply because of their size and no significant trapping interaction. However, the “soaking” of the shell with lighter hydrocarbon will change how NCS will interact with water and oil phases. Results indicated that the selective adsorption can be responsible for breakdown of the oil film. The proof comes from the continuum reduction in potential energy for the shell layer with hydrocarbons penetrating inside. (Fig. S13) A 300 ps running average of potential energy calculated using COMPASS force field is provided to show gain in PE as more molecules are adsorbed inside from the oil phase. The chemical potential or Gibbs free energy change for the absorption process is determined by the free energy of a small molecule in the oil film *vs* the hydrocarbon molecule inside the PS-b-PAA shell. The higher cohesive interaction inside the shell (shown in PE plot, Fig. S13) and relatively free torsional/vibration motion can make the NCS particularly adsorbent. Of course, the timescale available to MD is too small (nanoseconds) to observe saturation of the shell layer. Further effort in loading the shell with hydrocarbon needs to be performed to answer the unresolved mechanistic issues related to oil-NCS and NCS-(sea)water interaction for two limiting cases- Case-I: PS-PAA shell is saturated with lighter hydrocarbon, and Case-II: the NCS surface is covered by larger hydrocarbons (>C12). This will be pursued in future along with free energy estimations for other polymer combinations, and are currently outside the limited scope of the manuscript.

In order to assess the carbonaceous composition of solid state NCS, we analyzed samples through Raman spectrometry and concluded that the core structure shared graphitic and diamond like properties similar to CNP-PCDA particles (G/D band ratio of 1.11) (Fig. S1). The polymer signals were not visible in the Raman spectra due to the robust signal from the CNP-PCDA core. Transmission electron microscopy (TEM) image of NCS in anhydrous state (Fig. 3c) were analyzed for particle size, morphology and shell characteristics. Upon inspection, the NCS shells were clearly visible with a darker outer ring which is presumed to compose tightly compacted acrylic acid residues followed by a lighter inner ring consisting of low density styrene groups. Image J analysis of TEM images were used to quantify the size and ring thickness of anhydrous NCS particles (total count: 146 particles). This analysis revealed that the anhydrous particle diameters (D_ah_) were 109 ± 21 nm with the dark ring having a thickness of 13 ± 5 nm, and the light ring having a thickness of 13 ± 5 nm, with the core diameter being 29 ± 13 nm. The NCS size is much larger that the CNP-PCDA particles (D_hy_ = 8 nm) as predicted due to multiple particle packing within NCS core. The total TEM particle size analyses were found to be slightly smaller than D_hy_, presumably due to the flattening of bushy polymer chains and loss of water from hydration layer around NCS on TEM grids and attributed to the decrease of D_hy_ during the dehydration process. Tapping mode Atomic Force Microscopy (AFM) images were captured for NCS particles drop deposited over freshly-cleaved mica surfaces to investigate surface topography. Figure 3d showed that AFM measurements produced an average height (H_av_) of 111 ± 25 nm. A subsequent edge deflection image derived from AFM analysis showed a pattern of slope increases followed by slope decreases, suggesting the expected spherical morphology (Fig. 3e). Scanning Electron Microscopy (SEM) images were acquired on gold sputter-coated NCS samples at various magnifications to investigate NCS morphology (Fig. 3f, lower magnification images found in Fig. S4). This further revealed the spherical traits and surface topography of NCS particles. The complex multi-shell architecture of NCS and corresponding CNP-PCDA was further probed using wide-angle X-ray scattering (WAXS) and small angle X-ray scattering (SAXS)^51^ to obtain diffractogram of the shell structure. WAXS and SAXS analysis showed additional crystalline patterns on CNP-PCDA particles compared to NCS. D-spacing analysis can be found in the Supplementary Information (Fig. S5, Table S1). These physical characterization techniques offer enough information to predictable reconstruct a multi-shelled sphere as the prevalent theoretical structure for NCS (Fig. 3g).

Energy-dispersive X-ray spectroscopy (EDX) on both the NCS and CNP-PCDA revealed elemental integrity of both formulations with a significant increase in the oxygen abundance post polymer coating (Fig. S6). This observation can be explained due to the addition and subsequent abundance of oxygen-rich functionalities from PS-b-PAA polymer. UV-Vis absorbance analysis on NCS and its component groups showed a marginally shifted peak due to the presence of CNP-PCDA λ_max_ 283–293 nm and appearance of an additional peak at λ_max_ 222 nm characteristic of the styrene functional groups in the block polymer after polymer coating (Fig. S7). Finally, the electrophoretic potential (ζ, Zeta) of CNP-PCDA particles and NCS showed a highly negative surface charge (−33 ± 5 mV to −40 ± 6 mV respectively) predicting high colloidal stability^52^ (Fig. 3h).

### Quantification of NCS treatment efficiency on crude oil contaminated water

**Figure 4:**
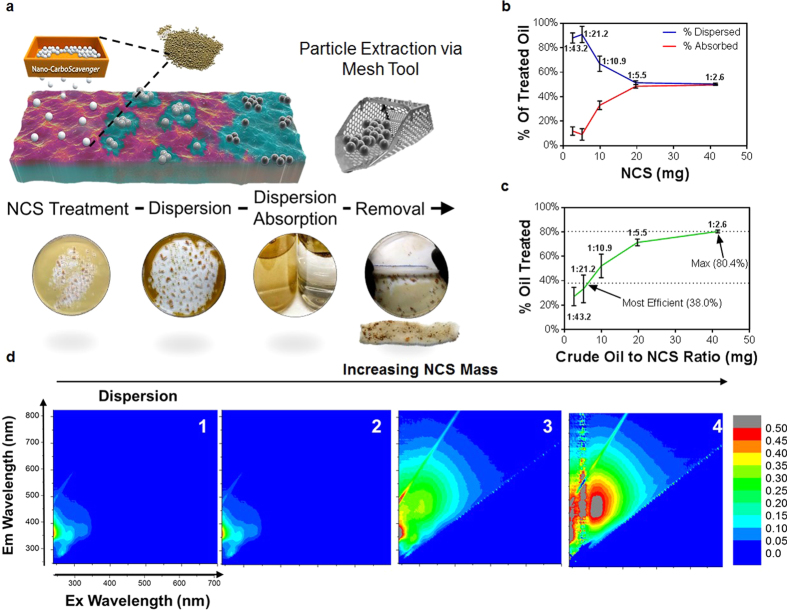
Quantification of NCS dispersion efficiencies on crude oil. (**a**) Graphic and live images depicting NCS powder distribution and the four steps of the scavenging process across oil compromised ocean water. At first interaction dispersion of oil in the water column is apparent while prolonged wave agitation leads to increased contact with petroleum and subsequent petroleum absorption (in live images, dispersion is highlighted with a green circle while clumping is indicated by an arrow). Particle extraction is performed using a mesh tool to remove spent particle from the water’s surface. Visual image of clump being extracted via a mesh tool can be found in Video 3. (**b**) Percentage of crude oil treated by dispersion (blue) and absorption (red) as a function of mg NCS used. (**c**) Percentage of total crude oil treated by NCS as a function of the ratio of mg crude oil to mg of NCS used. All point ratios in ‘**b**’ and ‘**c**’ represent treatment ratios of mg NCS to mg of crude oil. Experiments (**b,c**) were performed in triplicates for error bars of one standard deviation. (**d**) EEM Fluorescence images of dispersed crude oil in residual water with increasing mass of NCS treatment (left to right). (1) 0 g/L, (2) 1 g/L, (3) 4 g/L, (4) 8 g/L.

Our strategy for assessing oil spill treatment efficiency involves visual confirmation of NCS activity and quantitative mass analysis of oil sequestration and dispersion pre and post treatment of NCS on contaminated water samples. To calculate treatment efficiencies, NCS powder was spread over 108 mg of Saudi Arabian medium crude oil contaminated water (Full composition in Fig. S8, Purchased from ONTA) (surface area: 300 mm^2^) at NCS:oil mass ratios of 1:2.6, 1:5.5, 1:10.9, 1:21.2, and 1:43.2. As expected, the immediate dispersion effects were profound, and shortly followed by an additional entrapment effect (Video 3), (Fig. 4a). Samples were then placed on a nutator/rocking platform at 5 rpm for two hours to resemble the agitation caused by turbulent wave movement in oceans. The NCS crude oil absorption began to take place after extended interactions during wave action on the rocking platform. Additional oil stayed dispersed and suspended in the water column due to the rough agitation caused by the rocking platform (Fig. 4a) (Video 4). In all samples NCS darkened and accumulated into a floating clump for easy recovery *via* a gauze strip after treatment to resemble mesh collection used in large scale remediation (Fig. 4a). The container, NCS clump, gauze strip, and residual water were thoroughly mass balanced to quantify absorption and dispersion of crude oil (tests were performed in triplicates). NCS recovery *via* a gauze strip (Video 3) unveiled a collection efficiency of 84.3%. This recovery percentage did not take into account the oil dispersed into the water column for bioremediation or human error. Residual water with dispersed and solubilized contaminants was analyzed using Total Organic Carbon (TOC) to measure the amount of organic carbon present post NCS treatment. In order to exclude any solubilized NCS in TOC measurements, a measurement was conducted by TOC analysis on 50 mg NCS tumbled in 100 ml deionized water for 12 hours. An iterative mass balance revealed that NCS’s maximum solubility in deionized water is 0.02 g/L. Both, absorption and dispersion mass balances were combined in Fig. 4b to analyze efficient results. We observed that dispersion was dominant at lower ratios of oil to NCS, while absorption was dominant at higher ratios. We believe dispersion may have been dominant immediately after NCS powder distribution of all ratios (Fig. 4a, Video 4), but absorption was more prominent after extended interaction during the two hour simulated wave agitation incubation period (Video 3). The final reading showed the dispersed crude oil absorbed within the water column where the highest NCS treatment efficiency used 2.8 mg NCS to treat 41.3 ± 8.9 mg of crude oil (1:14.5 w/w ratio) for a total treatment percentage of 38 ± 8.5% as calculated by linear regression, while 80.4 ± 1.2% was the maximum percent of oil treated with a treatment ratio of 1:2.6 (mg NCS to mg crude oil) (Fig. 4c). An intensity quantification of excitation emission (EEM) fluorescence on residual water further revealed that crude oil dispersion increases with a higher NCS mass treatment (Fig. 4d, Fig. S9).

**Figure 5:**
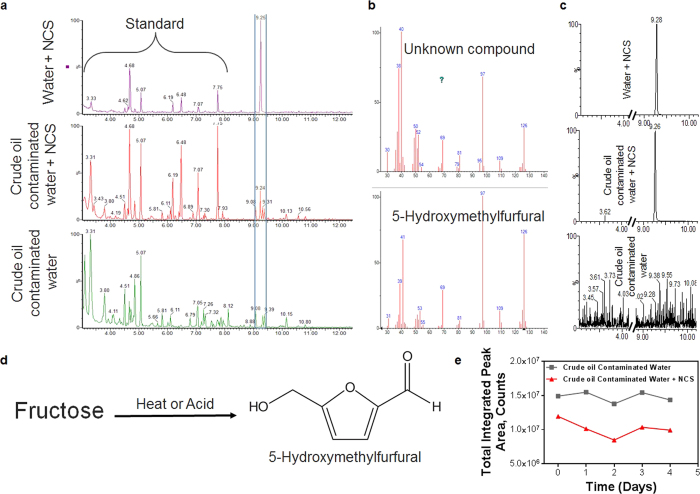
GC-MS analysis of water fraction after contamination with crude oil and treatment *via* NCS. (**a**) GC-MS plot (taken at mass 57.0704 m/z) of the water fraction from three treatment variations. The highlighted peak is associated with NCS presence. The ‘standard’, common among all samples, is contained in brackets. (**b**) Comparison of unknown NCS component to known peak signature for 5-Hydroxymethylfurfural’s (HMF). (**c**) High-resolution GC-MS analysis of unknown NCS component at mass equal to the molecular weight of HMF (126.03169 m/z). (**d**) Basic schematic representation of HMF synthesis and HMF structure. (**e**) Chart of integrated peak area **a,** comparing the NCS scavenging ability over four days.

To better understand the kinetics of the NCS structure during oil scavenging a Gas Chromatography-Mass Spectrometry (GC-MS) chromatogram study was performed over several days to analyze whether NCS components break down and release into the treated water. Three samples were prepared and tested in triplicate over four days of constant agitation and are as follows: NCS treated water, NCS treated crude oil contaminated water, and crude oil contaminated water alone. Each sample was extracted in organic solvents and measured at mass 57.0704 m/z (Fig. 5a). We found that GC-MS of NCS treated water, produced a signal for 5-(Hydroxymethyl)-2-furfural (HMF) (Fig. 5b), a non-toxic byproduct of heating sugar found in many food sources^53^,^54^, which was further confirmed by high-resolution GC-MS at 126.0317 m/z (Fig. 5c). Only the HMF peak was common among NCS treated crude oil contaminated water samples, but not in the crude oil contaminated water alone, indicating that no other signal was incident of NCS during crude oil treatment. This concludes that during treatment, the NCS-petroleum interactions do not lead to additional component release into the water column. Additionally, over the four-day treatment period, we observed a general trend of decreasing alkane groups solubilized within the water fraction as measured by peak sum at mass 57.0704 m/z (Fig. 5d). Thus, the NCS is functional over multiple days of treatment while efficiently absorbing free alkane groups.

**Figure 6:**
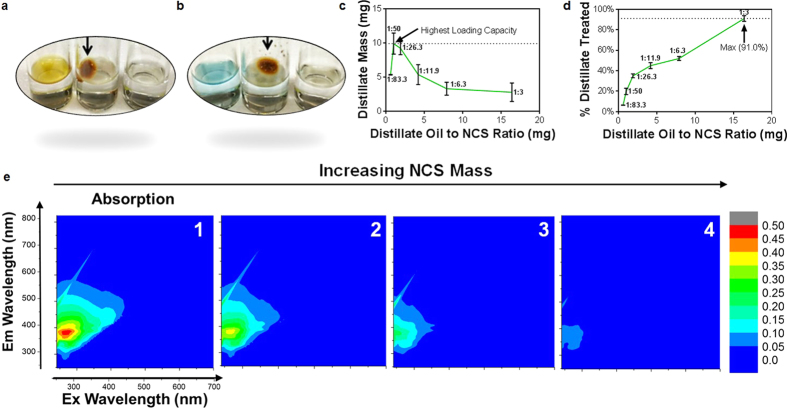
Quantification of NCS absorption efficiencies on a petroleum distillate. (**a**) Visual treatment of NCS absorption of distillate. (**b**) Visual treatment of NCS absorption of gasoline (dyed blue). In both (**a** and **b**), arrow addresses NCS clumping post absorption. (**c**) Loading capacity of NCS measured by mg distillate absorbed per ratio of mg distillate to mg of NCS used. (**d**) Percent of total petroleum distillate treated by NCS as a function of the ratio of mg distillate to mg of NCS used. All point ratios in **c** and **d** represent treatment ratios of mg NCS to mg of distillate oil. Experiments (**c** and **d**) were performed in triplicates for error bars of one standard deviation. (**e**) EEM Fluorescent images of solubilized distillate oil in residual water with increasing mass of NCS treatment (left to right): (1) 0 g/L, (2) 1 g/L, (3) 4 g/L, (4) 16 g/L.

Another form of crude oil component at risk of environmental spillage is its corresponding petroleum liquid distillate mixtures, such as gasoline, naphthalene, kerosene etc^55^. Thus we chose to test NCS efficiency on a petroleum distillate mixture composed of small chained liquid hydrocarbon varieties as investigated in the above MD simulation. A light Louisiana distillate mixture was added to water and treated by NCS following the same method used for crude oil treatment (Full composition in Fig. S10, Purchased from ONTA). NCS powder was distributed over 50 mg of distillate on water at NCS:distillate mass ratios of 1:3, 1:6.3, 1:11.9, 1:26.3, 1:50, and 1:83.3. Momentarily after treatment, the NCS formed a clump after absorbing the surrounding oil layer (Fig. 6a and b, Video 3) which was extracted for mass quantification. The container, NCS clump, and residual water underwent a thorough mass balance in order to measure the NCS absorption capabilities of distillate oil. This analysis unveiled that the maximum NCS loading capacity was 9.9 ± 1.5 mg of distillate per mg of NCS (Fig. 6c) when used at a treatment ratio of 1:50 (mg NCS to mg distillate), while the maximum distillate remediated was 91.0% when treated with a 1:3 treatment ratio (mg distillate to mg NCS) (Fig. 6d). An intensity quantification of EEM fluorescence on residual water further revealed a decreasing trend in solubilized hydrocarbon residues which indicated a true removal of surface and subsurface contaminants (Fig. 6e, Fig. S11). To further test NCS absorption capabilities on short-chained liquid hydrocarbons, gasoline was considered as a pure form distillate for its high concentration of octane groups^56^. Gasoline was mixed with AmeriColor™ blue oil dye (Indigo Carmine) (Structure can be found in Fig. S12) to visualize changes on the water surface. The resulting treatment visually demonstrated gasoline absorption with expected clumping (Fig. 6b). Hitherto, all samples were reproducible on 8 wt% salt water to mimic seawater salinity. These results were expected since NCS treatment of floating hydrocarbons happens at the water-surface interface and not in a water column.

### Assessing the biocompatibility of NCS

To investigate the biocompatibility of NCS, *in vitro* and *in vivo* experiments were designed utilizing a human cell line, a toxin-sensitive bacterial strain, and a fish species where each was exposed to various concentrations of NCS in culture media at 0.5, 1, 2, 4, and 8 g/L. To understand the scale at which unrecovered NCS would dwell in a surrounding aqueous environment, we assume a localized expected NCS exposure (LENE) to be maximally 1 × 10^−5^ g/L during petroleum spill remediation.

**Figure 7:**
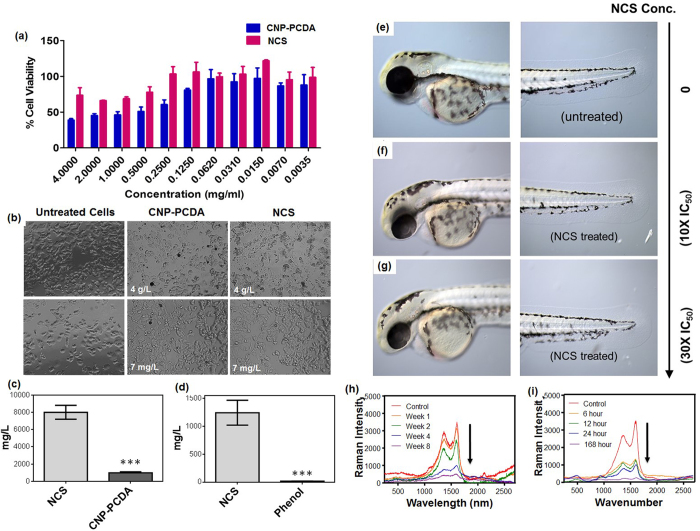
*In vitro* exposure of NCS to the human cell line MCF-7, the bacterial culture vibrio fischeri, and Zebrafish to quantify biocompatibility. (**a**) Histogram of percent difference in cell viability from control after CNP-PCDA and NCS exposure. Cell viability was measured *via* MTT assay in triplicates with a 10,000 cell density sample. (**b**) Bright field images of morphological and growth density variations in MCF-7 cell culture. (**c**) IC50 values calculated from MTT triplicate assay with error bars dictating one standard deviation. Stars reveal high statistical significance (student t-test, p-value <<  0.001). (**d**) Microtox EC50 Values measured in triplicates with error bars dictating one standard deviation. Stars reveal high statistical significance (student t-test, p-value <<  0.001). (**e**) Bright field image of zebrafish control post dechorionation (**f**) Bright field image of dechorionated zebrafish post in ovo treatment with NCS exposed egg water at 10x the calculated IC50 value. (**g**) Bright field image of dechorionated zebrafish post in ovo treatment with NCS exposed egg water at 30× the calculated IC50 value. (**h**) Raman overlay analysis of NCS exposed to HMPO over a 168-hour period. (**i**) Raman overlay analysis of NCS exposed to HRP over an 8-week period. Raman analysis operated at 2% laser power for 1 min with a 532 nm laser.

A 2D-culture of human estrogen receptor positive cells (MCF-7) was treated with NCS to evaluate exposure on human cells (Fig. 7a,b). Cytotoxicity was measured by MTT reduction assay using a plate reader (Synergy HT, Bio-Tek). Cell viability was measured as percent difference from control cells to calculate IC_50_ and was found to be 8 g/L for NCS and 1 g/L for CNP-PCDA particles (Fig. 7c). Variations in cell viability comparing CNP-PCDA and NCS are considered biologically significant with a p-value less than 0.001. Although these concentrations greatly surpass LENE concentrations, the results indicated an eight-fold improvement in cell viability upon surface protection of single shelled CNP-PCDA particles.

A Microtox bacterial assay^57^,^58^,^59^ (Environmental M500 Microtox analyzer) provided acute toxicity information on NCS through bioluminescent quantification of the bacterial strain *Vibrio fischeri*. Bacterial cultures were exposed to NCS concentrations 0.5, 1, 2, 4, and 8 g/L, then subsampled and exposed to bacterial cultures at 0%, 9%, 36%, 63%, and 90% for 5 minutes, and measured for luminescent intensity. The effective NCS concentration which produced a 50% decrease in luminescent intensity (EC_50_) was calculated by linear regression and found to be 1.28 g/L (Fig. 7d) compared to a phenol test which gave an EC_50_ of 0.02 g/L. Variations in bacterial viability comparing CNP-PCDA and NCS are considered biologically significant with a p-value less than 0.001.

Zebrafish (*Danio rerio*) were chosen as a model organism for investigating aquatic vertebrates for NCS exposure due to their environmental sensitivity and transparent characteristics for internal organ analysis^60^,^61^,^62^. The egg water^63^ was exposed to various concentrations of NCS for 24 hours. Five sets of 15 wild type embryos 26 hours post fertilization were then treated with 5 ml of NCS exposed growth media for an additional 24 hours prior to the expected hatching period in order to analyze developmental effects during *in ovo* exposure. NCS particles did not exhibit any detrimental damage on the development of zebrafish embryos until concentrations far exceeded LENE concentrations. At an extremely high concentration, some toxicity-related symptoms were observed (Fig. 7e–g) possibly due to solubilization of NCS as calculated before to be maximally 0.02 g/L. The main observed defects in the highest concentration were: delay hatching, edema, blood pooling, slowing heartbeat, small eye, fin fold malformations reduce response to touch stimulation and embryo death. LD_50_ was indicated when 50% of embryos expressed severe symptoms and was calculated to be 4 g/L. For comparison, the EPA reports Corexit EC9500A, the dispersant used at the Deepwater Horizon oil spill, has an LC50 of 0.0252 mg/L or 25.2 ppm^64^ (Table 1). For a robust review and comparison of NCS to current dispersants and nanomaterials regarding treatment efficiency and toxicity, see Supplementary Table S2).

### Enzymatic Degradation of NCS

To further establish NCS bio-compatibility, we considered direct uptake and interaction with surrounding flora and its incidental degradation by the plant based enzyme horse radish peroxidase (HRP). Five samples containing a suspension of 1 mg/ml of NCS and 0.5 mg/ml HRP were statically incubated in 5 ml of Dulbecco’s phosphate buffered solution (dPBS) and 80 μM of hydrogen peroxide at 4 °C in the dark to mimic *in vivo* conditions. Individual samples were removed at selected intervals of 1, 2, 4, and 8-week time points and drop-casted onto glass slides to prep for Raman analysis to visualize enzymatic degradation on the molecular level^65^. After graphical analyses, we found a significant decrease and near disappearance of graphitic (G) and diamond (D) bands after extended exposure to enzyme indicating degradation and sp2/sp3 carbon bond oxidation of the carbon core (Fig. 7h). The control sample followed the same procedure without enzyme and did not show change in G/D band intensity after an 8-week incubation period, thereby ruling out self-degradation or destruction *via* media. Due to the strong carbonaceous nature of NCS, PS-b-PAA and PCDA peaks were not visible during Raman spectroscopy.

Accidental exposure to humans is entirely possible if NCS finds its way to an aqueous stored food supply; hence, an analysis on NCS digestion by enzymes abundant in mammalian system was necessary. We evaluated this type of degradation of carbon-based material by a common human digestion enzyme, human myeloperoxidase (HMPO)^66^. Five suspensions of 1 mg/ml NCS in phosphate buffer DPBS and 200 μM of hydrogen peroxide were prepared to mimic *in vivo* conditions for enzymatic digestion. 100 μg of HMPO enzyme (activity >50 units/mg protein, Sigma) was added to four of the five samples and then statically incubated at 37 °C in the dark. Samples were collected at 6, 12, 24, and 168 hours and drop-cast onto glass slides for Raman analysis. Similar to the HRP degradation, we found that both the G and D band significantly decreased in intensity indicating a dramatic change in carbon core sp2 and sp3 bond morphology (Fig. 7i). The control sample was incubated for 168 hours in similar conditions without the enzyme and did not show a change in G/D band intensity, thereby ruling out degradation *via* media. Interestingly, the enzymatic degradation of NCS carbon core using HMPO was found to be much faster (days) in comparison to HRP, which required weeks to observe complete dissociation.

## Conclusion

Ecosystem dependent bodies of water are still at risk of severe petroleum contamination as seen with the 90,000 gallon Shell oil spill recently in May, 2016^67^. Current methods to salvage such situations involve commercially mass-produced chemical “dispersants” applied to blossoming oil slicks to hinder migration of the spillage towards coastal areas. However, increasing numbers of independent recent studies show that the chemical dispersants are not only toxic to marine life, but also cause increased toxicity when pooled with crude oil. While long term effects of these chemicals are still unknown, their acute toxicity to marine and land organisms led us to develop a transformative technology. Our approach provided a nanotechnology-based biocompatible platform for remediating crude oil and distillate spills on various types of aqueous environments. In simulated micro-spills, the NCS were found to rapidly disperse and consecutively absorb crude oil contamination with prolonged wave agitation. The NCS were found to efficiently absorb a petroleum distillate with a high loading capacity compared to current nanomaterials. In all cases, the carefully designed NCS nano-architecture allowed for material clumping post treatment of oil contaminated waters for simplified recovery *via* mesh nets or booms on large scales without leaving residual signatures in the aqueous environment, as tested with a simulated mesh gauze strip. In order to anticipate less than 100% nanomaterial recovery, we have extensively considered the potential interactions and effects of NCS on biological organisms. Primarily, the functional aspect of the NCS is that it is intended to be removed from the surface of the water with an ideal 0% footprint. We understand that this may not be a realistic scenario, therefore we have taken measures to refute concerns regarding any potential environmental risks. For example, our GC-MS analysis of residual water did not detect any of the characteristic signatures caused by PS-b-PAA post scavenging process. It indicated that there was no detectable amount of PS-b-PAA left in scavenged water. Further development towards the next generation nano-carboscavengers that we are currently investigating have moved towards different polymer molecules with significantly high biodegradability. NCS were found to be remarkably un-inhibitive when exposed to *V. fischeri*, MCF-7, and zebrafish until LENE concentrations were far exceeded. This observation was further bolstered as the carbonaceous nature of the NCS system was digested by a common plant and human enzyme. Overall effectiveness surpassed other nanoparticle-based treatments, as confirmed by TOC analysis, fluorescence studies, and other analytical means, making the NCS a superior substitute to current nanotechnologies. Future studies will expand on particle functionality in diverse environments on and off shore including remediating land spills and purifying fracking waters. Since NCS are derived from natural carbohydrate sources, the calibrating particle structure and passivating agents with high nitrogen and phosphorous content can extend NCS capability as a bioaugmentation supplement to hydrocarbon degrading bacteria during oil spill remediation^68^. Hence, this polymer passivated nanomaterial used for crude oil absorption and solid state dispersion offers a primary foundation for oil spill removal and has proven to be a highly efficient, multi-functional pollution remediating nanomaterial.

## Methods

Unless stated otherwise, all material and reagents were purchased from Sigma–Aldrich, St. Louis, MO, and were used without further purification. Solvents were used as received and without further purification.

### NCS Synthesis

The CNP-PCDA core of the NCS was produced by a hydrothermal method of controlled heating in a suspension of 250 mg/ml of nectar agave (HoneyTree’s Organic Agave Nectar, Onsted, MI) and 37.5 mg/ml of pentacosadiynoic acid (Sigma-Aldrich, St. Louis, MO) in nanopure water (0.2 μm) at 250 degrees Celsius for 45 minutes or until all water evaporates and product turns black mass. This residue was then soaked in THF overnight then resuspended by sonication (Q700, Qsonica Sonicators, Newtown, CT) in an ice bath (to prevent evaporation) for eight minutes at one amplitude with intervals of five seconds on and three seconds off. The suspension was filtered through 0.45 μm and 0.22 μm PTFE membranes. The suspension was dried out to produce a residue. This residue and (PS_275_-b-PAA_50_) at a 10:2 mass ratio respectively, were resuspended in THF. The volume of THF used is the minimum required to fully dissolve solutes. Water was then added rapidly at twice the volume of THF to produce the block polymer coated nanoparticles known as the NCS. This suspension was placed in a vacuum to dry and then scraped and ground into powder form.

### Investigating the PCDA crosslinking onto carbon core

In a 20 mL scintillation vial, agave nectar (HoneyTree’s® Organic Agave Nectar, Onsted, MI) and 10,12-pentacosadiynoic acid were mixed in water (4 mL) at concentrations of 250 mg/ml and 37.5 mg/ml, respectively. The sample was bath sonicated (Branson 1510) for 10 minutes at room temperature and then mechanically mixed (Benchmark Bench Vortex Mixer). The agave and PCDA that did not dissolve was separated from the suspension. The scintillation vial was then placed on a hot plate at 250 °C and samples were collected at 0, 4, 5, 6, 8, 9, and 10 minutes. The crosslinking was monitored using a UV-VIS Spectroscopy (GENESYS 10 S). To measure the collected samples, a 1 to 20 dilution of the heated sample was prepared in deionized water for each UV-VIS measurement where a stock solution agave in water at the same concentration was used as a blank.

### Oil scavenging efficiency quantification

For dispersion quantification of heavy crude oil we used Saudi Arabian medium-heavy crude oil (2.48% sulfur by weight, API gravity of 31.1, viscosity at 19 mm^2^/s at 25 °Celsius, ONTA). 120 microliters of the crude oil was pipetted onto 50 ml of carbon filtered water (surface area 300 mm^2^). The crude oil surface layer was treated with NCS and the container was then placed on a rocking platform (VWR) at 5 rpm for two hours. The NCS aggregates were removed and weighed while the water layer was extracted from the crude oil layer for TOC analysis.

For NCS absorption quantification of petroleum distillates, we purchased a Louisiana distillate sample (0.14% Sulphur by weight, API gravity 43.2, ONTA) with traces of gasoline and naptha at 32.2%, kerosine at 16.3%, gas oil at 11.4%, lubricating distillates at 14.1%, and residuum at 26%. Fifty microliters of the distillate sample was pipetted onto the surface of 10 ml of water. The sample was then treated with NCS at varying masses. The clump formed by the CarboScavenger was removed and weighed, while the extracted water was analyzed by TOC.

Total organic carbon analysis was performed at the Illinois Sustainable Technology Center. Carbon measurements were performed with a Shimadzu TOC-V analyzer. The instrument was operated in non-purge-able organic carbon mode with addition of 2 M hydrochloric acid and a spurge time of 1.5 minutes. The instrument was calibrated before use by serial dilutions of a potassium hydrogen phthalate stock solution over a range of 1 mg/L to 100 mg/L. Three to five injections were made on each sample with a reproducibility target setting of 0.1 maximum standard deviation.

Fluorescent measurements were performed at the Illinois Sustainable Technology Center to measure solubilized hydrocarbons within water column. EEM measurements were taken with a Horiba Aqualog combined scanning spectrophotometer and spectrofluorometer. The samples were scanned over an excitation range of 240 nm to 700 nm and an emission range of 246 nm to 828 nm. The integration time was set at 0.1 seconds and the wavelength scan increments were set for 3 nm and 5 nm, excitation and emission, respectively. All samples were blank subtracted with data obtained from analysis of a deionized water blank. Post processing of the fluorescence data included inner-filtering correction with data obtained by simultaneous absorbance measurements and Rayleigh masking of signals produced as a result of light scattering of water. In addition, all emission intensities were normalized to a 1 mg/L quinine sulfate solution that was analyzed prior to each assay.

### Surface tension quantifications

A Ramehart goniometer equipped with a Ramehart camera and lighting system was used for imaging and analysis. A 22-gauge syringe connected to a Teflon capillary was used to supply a drop of liquid into the viewing area. All liquid-surface angle data was measured on parafilm. All experiments were performed at room temperature. The maximum contact angles were measured every 0.5 seconds for 30 repetitions carefully around the drop and subsequently averaged.

### Gas Chromatography Mass Spectrometry Analysis

One of each triplicate or duplicate sample from each time-point was selected for extraction. Fifty ml of the sample was transferred to a 250 ml separatory funnel and 0.05 ml of a stock p-Terphenyl-d14 surrogate was added to each sample to evaluate the sample extraction and to monitor any losses. Each sample was then extracted with 100 ml methylene chloride and twice with 100 ml hexane. The organic fraction was then pooled, exchanged to hexane, and concentrated to a final volume of 1.0 ml with TurboVap concentration units operated at 40 °C. Note that this extraction is a non-polar extraction and only compounds soluble in dichloromethane and hexane would be extracted by this procedure.

The extracts were analyzed with an Agilent HP6890 gas chromatograph coupled to a water AutoSpec Ultima mass spectrometer. A 1 μl aliquot of the extract was injected into the instrument at 300 °C and with a split ration of 5:1. Separation was achieved on an Agilent J&W DB-XLB capillary column (30 m × 0.25 mm × 0.25 μ df) with a 5 m deactivated guard column. Helium at a flow rate of 1.0 ml/min was used as the carrier gas. At the time of injection, the oven has held at 70 °C for 1.5 minutes. After that time the oven was ramped to 340 °C at a rate of 10 °C/min. Once a temperature of 340 °C was reached, it was held there for 10 minutes. For general scan assays, masses were collected from 30 to 350 mass to charge (m/z). Post data processing included mass calibration using perfluorokerosene (pfk) and blank subtraction within the MassLynx software. High resolution measurements also utilized the appropriate pfk fragment for lock mass depending on the high resolution mass measured.

### Enzymatic digestion of NCS

To evaluate the NCS integrity in the presence of HRP, one mg/ml of NCS was suspended with 0.5 mg/ml HRP (Thermo Scientific) in 5 ml of Dulbecco’s phosphate buffered solution and in an excess of 80 μM of hydrogen peroxide and was then statically incubated at 4 °C in the dark as previously reported^69^. At the one-week, two-week, four-week, and eight-week time points, the suspensions were removed from incubation, centrifuged at 10 k RPM for 30 seconds and the supernatant was replaced with nanopure water to halt any residual enzyme reaction. The suspension was drop cast onto glass slides for Raman analysis (120 to 2700 nm at 0.2% laser power for 60 seconds).

To evaluate the NCS integrity in the presence of HMPO, our methods involved making five suspensions of 1 mg/ml NCS in Dulbecco’s phosphate buffered solution and 200 μM of hydrogen peroxide. One hundred μg of HMPO enzymes (activity > 50 units/mg protein) was added to four of the five samples and then statically incubated at 37 °C in the dark. Hydrogen peroxide was added every five hours to maintain a 200 uM concentration and to keep enzyme activity constant as previously reported^65^. The treated and control samples were incubated at 37 °C for 24 hours. At 6, 12, 24, and 168 hours, samples were removed from incubation and placed in 4 °C environment for 4 hours to halt enzyme activity. Samples were then collected and drop cast on glass slides for Raman analysis (120 to 2700 nm at 2% laser power for 60 seconds).

### Raman Spectroscopy

All Raman measurements were taken on a Nanophoton Raman instrument (Materials Research Building, University of Illinois Urbana-Champaign) with a 532 nm wavelength laser for one min at 0.2% laser power using a 20× objective. For each spectrum a grating (600 l mm^−1^) scan was taken over the range of 120–2700 cm^−1^. An average of 20 spectra was recorded and averaged for every sample.

### Dynamic Light Scattering Measurements

Hydrodynamic diameters were determined using a Malvern Zetasizer ZS90 particle size analyzer, while scattered light was collected at a fixed angle of 90°. A photomultiplier aperture of 400 um was used with the incident laser power adjusted to obtain a photon counting rate between 200 and 300 kcps. Diameter values depended on measurements whereupon the measured and calculated baselines of the intensity autocorrelation function had to agree within +0.1%. Hydrodynamic diameter was analyzed using number distribution in accordance with previous reports^70^. All determinations were made in multiples of three consecutive measurements with 15 runs each.

### Determination of Surface Zeta Potential

Zeta potential (ζ) values for NCS were determined with a nano series Malvern Zetasizer zeta potential analyzer. Data were acquired in the phase analysis light scattering (PALS) mode following solution equilibration at 25 °C when calculation of ζ from the measured electrophoretic mobility (μ) employed the Smoluchowski equation, which is expressed as μ = εζ/η (where ε and η are the dielectric constant and the absolute viscosity of the medium, respectively). Measurements of ζ were reproducible to within ±6 mV of the mean value given by three determinations of 15 data accumulations.

### UV-VIS Measurements

The absorption spectra for NCS and PCDA crosslinking were acquired in a Genesys 10 S UV–VIS Spectrophotometer (Thermo Fisher Scientific, Rockford, IL).

### Transmission Electron Microscopy Measurements

A drop of CarboScavenger suspension was placed on a carbon coated TEM grid. The TEM images were acquired on a JEOL 2100 Cryo TEM machine and imaged by Gatan UltraScan 2 k × 2 k CCD.

### Atomic Force Microscopy

A Digital Instruments Dimension 3000 series AFM (from MRL facility, UIUC) was used for scanning the samples using standard Veeco tapping mode silicon probes with a platinum–iridium (PtIr) coating. In a typical experiment, suspended NCS samples were dried in a desiccator on freshly cleaved mica. Dried samples were placed on the AFM platform and scanned. Pertinent scanning parameters were fixed as follows: Example of tip velocity: 4 m/s for 2 m, 15 m/s for 5 m, 30 m/s for 10 m; resonant frequency (probe): 60–80 kHz. Aspect ratio: 1:1; resolution: 512 samples/line, 256 lines 2.10.

### Scanning Electron Microscopy and EDX Analysis

Scanning electron microscopic studies were performed on coated NCS to evaluate surface properties of their clusters. Additional energy dispersive X-ray (EDX) spectrum analysis was also performed to demonstrate the presence of elements in addition to C and O in particle preparations. Samples were prepared on metal stubs decorated with Si block. Suspension of particles were used to drop cast on carbon tape and allowed to air-dry before putting in vacuum for 5 h.

SEM images were taken on a Hitachi S-4700 High Resolution SEM machine with capabilities of high resolution low voltage imaging and connected to a cold field emission gun (2.5 nm resolution at 1 kV, 1.5 nm resolution at 15 kV) while EDX was performed on ISIS EDS X-ray Microanalysis System (Oxford Instruments) used tp build software.

### Wide and Small Angle X-ray Diffraction Studies

WAXS/SAXS measurements were carried out on an X-ray diffraction machine with a Pilatus 300 K area detector (172 μm pixel). The Cu Kα radiation (λ = 1.54 Å) was collimated with an X-ray mirror and two pairs of scatter less slits and passed through the evacuated path of 1600 mm from source of Cu Kα radiation to sample holder. The 2D-patterns were recorded on an image plate and processed using software. All the measurements were made in the transmission mode. The sample to detector distance was 1400 mm for SAXS and 136 mm for WAXS, respectively. Powdered NCS and CNP-PCDA samples were used for the analysis.

### Biocompatibility assays

The experimental protocol was approved by the Institutional Animal Care and Use Committee (IACUC), Northwestern University Feinberg School of Medicine, and satisfied all University and National Institutes of Health (NIH) rules for the humane use of laboratory animals. All the animal experiments were carried out in accordance with the approved guidelines. All methods were carried out in accordance with relevant guidelines and regulations. All experimental protocols were approved by Northwestern University.

The growth media were exposed to concentrations of NCS (0.5, 1, 2, 4 g/L) for 24 hours. Five sets of 15 wild type embryos (ABTU hybrid line) 26 hours post fertilization were treated with 5 ml of NCS exposed growth media water for 24 hours. Embryo viability were then analyzed under a light microscope and imaged.

Microtox was performed at the Illinois Sustainable Technology Center. All the bacterial experiments were carried out in accordance with the approved guidelines. All methods were carried out in accordance with relevant guidelines and regulations. All experimental protocols were approved by Illinois Sustainable Technology Center, Prairie Research Institute, University of Illinois at Urbana Champaign.

The acute toxicity reagent, reconstitution solution, and diluent were obtained from Modern Water, Inc. (New Castle, DE). The phenol stock material (99.5% purity) was obtained from Alfa Aesar (Ward Hill, MA) and sodium chloride (99.9% purity) was obtained from Thermo Fisher Scientific (Waltham, MA). Sodium chloride was used to adjust the osmotic pressure of each test sample and reference material to approximately 2% NaCl. Each sample was assayed at 0%, 9%, 36%, 63%, and 90% concentrations. The measurements were performed with an AZUR Environmental Microtox®M500 analyzer (purchased from Modern Water, New Castle, DE). The test organism was incubated at 15 °C for 10 minutes before exposure. The exposure time was 5 minutes and light measurements were performed before and after exposure to test materials. The effective concentration which produces 50% maximum response, EC50, was calculated by linear regression to show the difference of pre-exposure and post exposure light emission versus concentration of test solution. A phenol reference material was also analyzed with each run as quality control. The known EC50 for phenol was conducted with a 5-minute exposure in the range of 13 to 26 mg/L56 for a valid assay of phenol reference material.

### MD Simulation Studies

The classical MD simulations performed use Forcite Plus under Materials Studio environment from BioVIA. The MD simulations were performed using a canonical ensemble method where number of particles, system volume and temperature are constant (NVT ensemble). For the MD simulations performed, COMPASS forcefield by Sun *et al*. was used to reproduce the polymer and hydrocarbon dynamics (*Sun, H.,“COMPASS: An Ab Initio Forcefield Optimized for Condensed-Phase Application-Overview with Details on Alkane and Benzene Compounds”, J. Phys. Chem., 1998, B102, 7338–7364*). The PS-PAA layer simulated was constructed in a 7 × 7 × 10.5 nm periodic box using experimental density of PS-PAA layer with a vacuum layer in-between. The hydrocarbon droplet was placed on the surface and the MD simulation was performed for 200 ps for equilibration followed by 300 ps for calculating averages. The COMPASS force field is well suited for polymer/water and polymer/hydrocarbon interactions. COMPASS was parameterized to reproduce the ab-initio conformational energies for simple model compounds. PS and PAA polymers have been studied using the force field and interactions with carbon nanotubes have been previously published with reasonable agreement in density and conformational dynamics (*Liu et al. J. Phys. Chem. C 2008, 112, 1803–1811*).

## Additional Information

**How to cite this article:** Daza, E. A. *et al*. Multi-Shell Nano-CarboScavengers for Petroleum Spill Remediation. *Sci. Rep.* **7**, 41880; doi: 10.1038/srep41880 (2017).

**Publisher's note:** Springer Nature remains neutral with regard to jurisdictional claims in published maps and institutional affiliations.

## Supplementary Material

Supplementary Information

## Figures and Tables

**Table 1:** Table comparison of nanomaterials, commercial powder absorbents, and commercial chemical dispersants with remediation ratio (agent to petroleum) and reported toxicity.

Type	Agent	Remediation Ratio (Agent to Petroleum)	Toxicity (ppm)		Ref. #
**Nanomaterial Absorption and Dispersion**	**Nano-CarboScavenger**	**1:14.75** w/w (Crude Oil) **1:9.9** w/w (Petroleum Distillate)	*V. fischeri* MCF-7 *D. rerio*	**1280 8000 4000**	—
Nanomaterial Absorption	Photoinduced Polymer Nanoparticle	750 mg:1 litre	*Not Calculated*	—	20
Nanomaterial Absorption	Hydrophobic Iron Core Nanoparticle	1:3.8 w/w	*Not Calculated*	—	25
Nanomaterial Absorption	Polymer - Iron Nanoparticle hybrid	1:10 w/w	*Not Calculated*	—	26
Absorption Powder	OIL SOLUTIONS POWDER	1:04 w/w	*M. beryllina M. bahia*	22.5 2.13	65
Chemical Dispersant	BIODISPERS	1:10 v/v (Crude Oil) 1:20 v/v (Pure Solvents)	*M. beryllina M. bahia*	13.46 78.9	65
Chemical Dispersant	Corexit9500 EC9500A	1:10 v/v	*M. beryllina M. bahia*	25.20 32.23	65

Remediation ratio is reported as weight per weight (w/w) or volume to volume (v/v) ratios. Toxicity for *M. beryllina* and *M. bahia* is reported as their respective LC_50_ values.
