# Supplementary material for: Multi-Shell Nano-CarboScavengers for Petroleum Spill Remediation
Source: Sci Rep. 2017 Feb 3;7:41880. doi: 10.1038/srep41880 (PMC5291094; doi:10.1038/srep41880)
Supplement: Supplementary Information [file srep41880-s1.pdf]

Supporting Information for:

Multi shell Nano-CarboScavengers for Petroleum Spill Remediation

Enrique A. Daza[1,2+], Santosh K. Misra[1,2+], John Scott[3], Indu Tripathi,[1,2] Christine Promisel[1], *Brajendra K. Sharma[3], Jacek Topczewski[5], Shantanu Chaudhuri[4], Dipanjan Pan[1,2]**

[1]Department of Bioengineering, University of Illinois at Urbana Champaign, Urbana, Illinois 61801.

[2]Carle Foundation Hospital, Urbana, Illinois, 61801.

[3]Illinois Sustainable Technology Center, Prairie Research Institute, University of Illinois at Urbana Champaign, Champaign, Illinois, 61820.

[4]Applied Research Institute, Champaign, Illinois, 61820.

[5]Department of Pediatrics, Northwestern University Feinberg School of Medicine, Stanley Manne Children's Research Institute, Chicago Illinois 60611,

[+]authors of equal contribution.

*Corresponding author: e-mail: dipanjan@illinois.edu

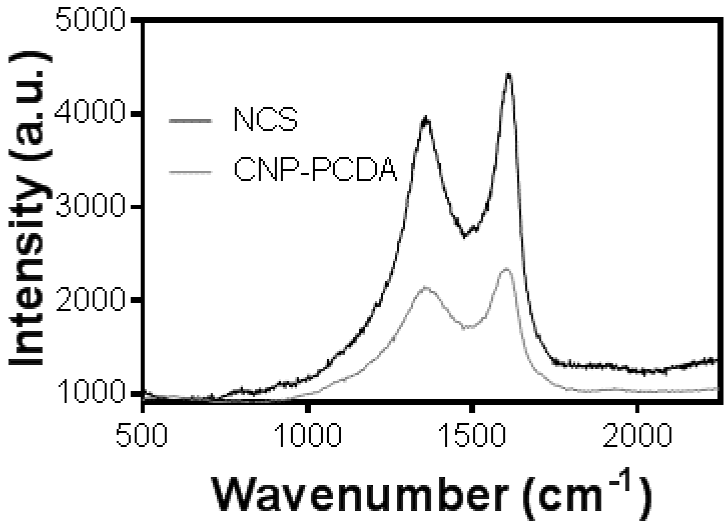

Figure S1 | Raman spectra showing the graphitic and diamond like characteristics of NCS and CNP-PCDA.

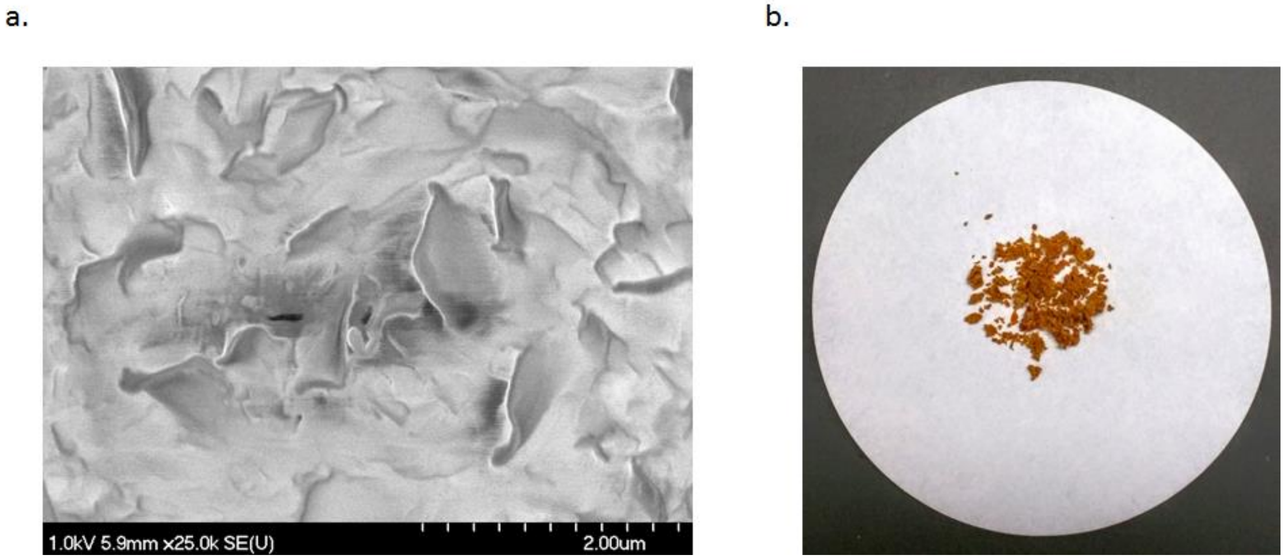

Figure S2 | (**a**) SEM image of CNP-PCDA film left after THF evaporation. (**b**) CNP-PCDA flakes after THF evaporation and collection.

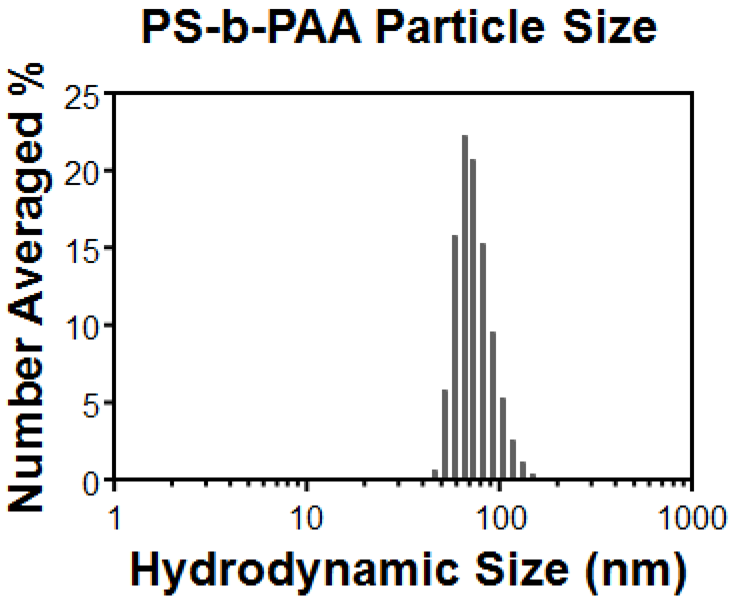

Figure S3 | Number averaged histogram depicting the hydrodynamic diameter of PS-*b*-PAA particles in water (75 ± 18 nm with a PDI of 0.16 ± 0.01) as measured by DLS.

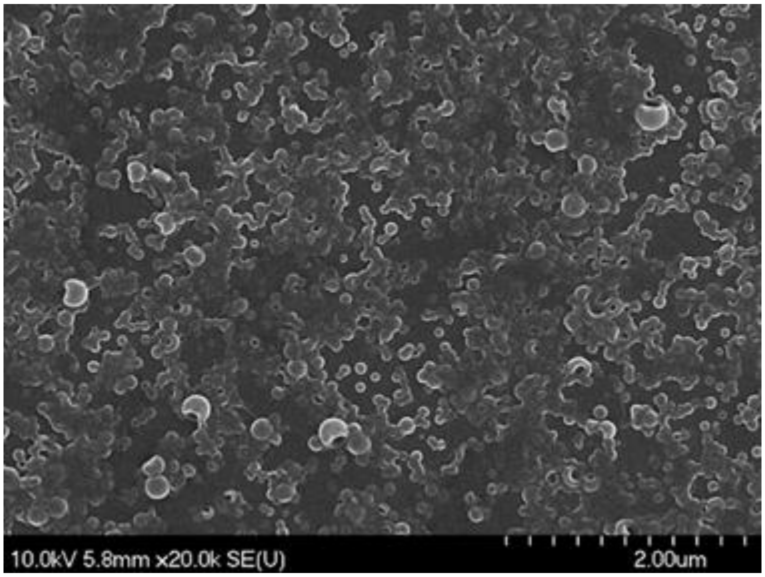

Figure S4 | Low magnification SEM image of anhydrous NCS on silicon.

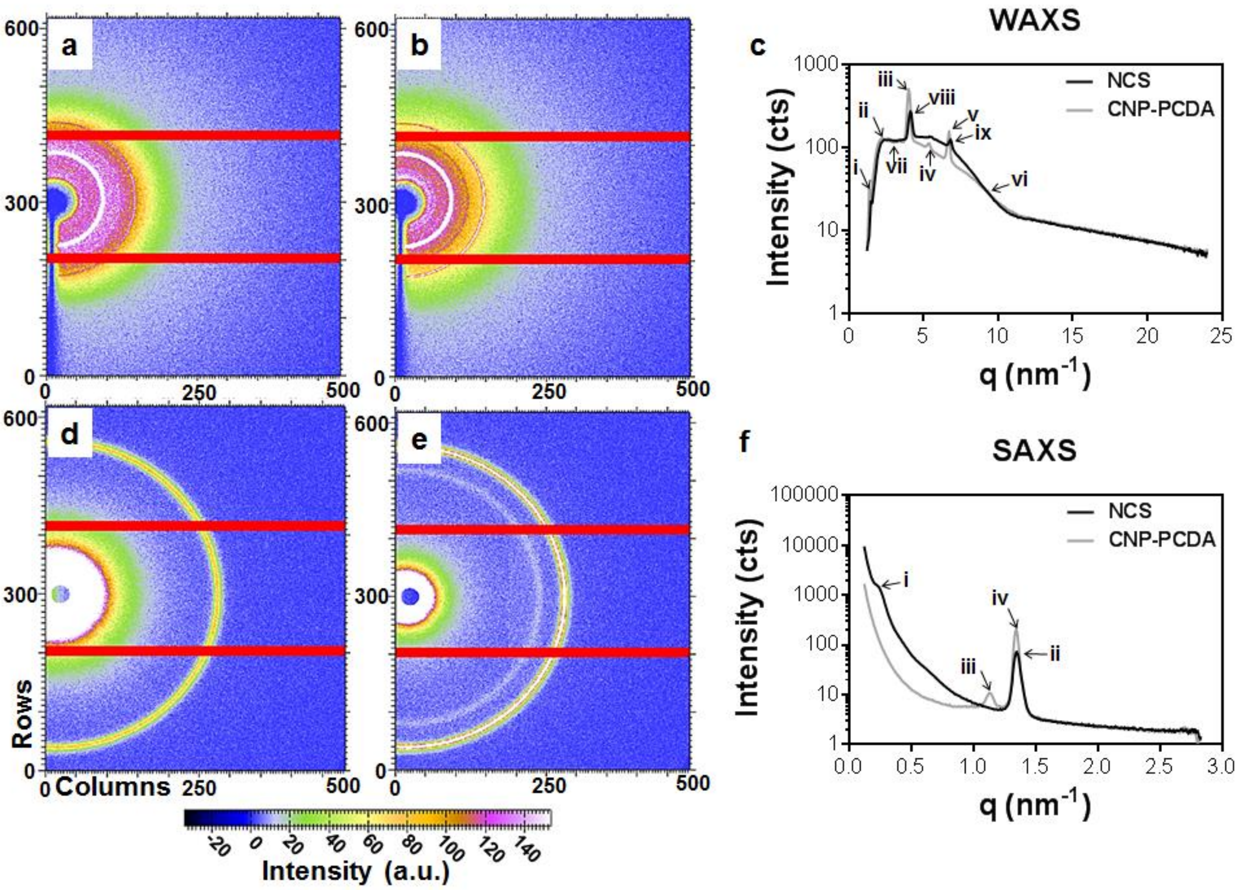

Figure S5 | Shell characterization, and compositional analysis of NCS and CNP-PCDA. (**a**-**c**) Wide angle x-ray diffraction (WAXS) pattern of **a** NCS and **b** CNP-PCDA with **c** overlay graph of variations in individual d-spacing. (**d-f**) Small angle x-ray diffraction (SAXS) patterns of **d** NCS and **e** CNP-PCDA with **f** overlay graph of d-space variations. These variations in d-spacing patterns correlate with change in surface coatings from CNP-PCDA to NCS.

	CNP-PCDA						NCS		
WAXS	i	ii	iii	iv	v	vi	vii	viii	ix
q (nm-1)	2.33	2.43	4.14	5.51	6.85	9.74	2.77	4.14	6.8
d nm	2.7	2.59	1.52	1.41	0.92	0.663	2.27	1.52	0.92
2θ (degrees)	3.28	3.42	5.82	7.75	9.64	13.35	3.895	5.82	9.57
SAXS	i	ii	*	*	*	*	iii	iv	*
q (nm-1)	0.23	1.352	*	*	*	*	1.14	1.352	*
d nm	27.2	4.65	*	*	*	*	5.51	4.65	*
2θ (degrees)	0.325	1.9	*	*	*	*	1.6	1.9	*

Table S1 | WAXS and SAXS peak identification of d-spacing variations. WAXS peak numbers refer to Fig. S5c. SAXS peak numbers refer to Fig. S5f.

WAXS and SAXS in depth explanation. The WAXS diffractogram revealed the presence of common layered architecture with d-spacings of 0.92, 1.52 and 1.15 nm in NCS and CNP-PCDA (Table S1). Other layered architectures with d-spacings of 2.7, 1.77 and 0.663 nm were present in the CNP-PCDA particles while NCS showed presence of 2.59 nm (Fig. S5c, Table S1). Further investigations of SAXS diffractograms revealed presence of common layered architecture with d-spacing of 4.65 nm which varied to 5.51 nm for the CNP-PCDA while coated NCS showed layered architecture of 27.2 nm d-spacing (Fig. S5f, Table S1). Variations in d-spacing of layered architectures of NCS and CNP-PCDA signified the changes in inner architecture of NCS particles. Appearance of 27.2 nm d-spacing in NCS is probably associated with polymer coating, compared to 5.51 nm for cross linked shell architecture from PCDA in CNP-PCDA particles (Table S1), which also corroborated with the findings from the TEM inset in Fig. 3a.

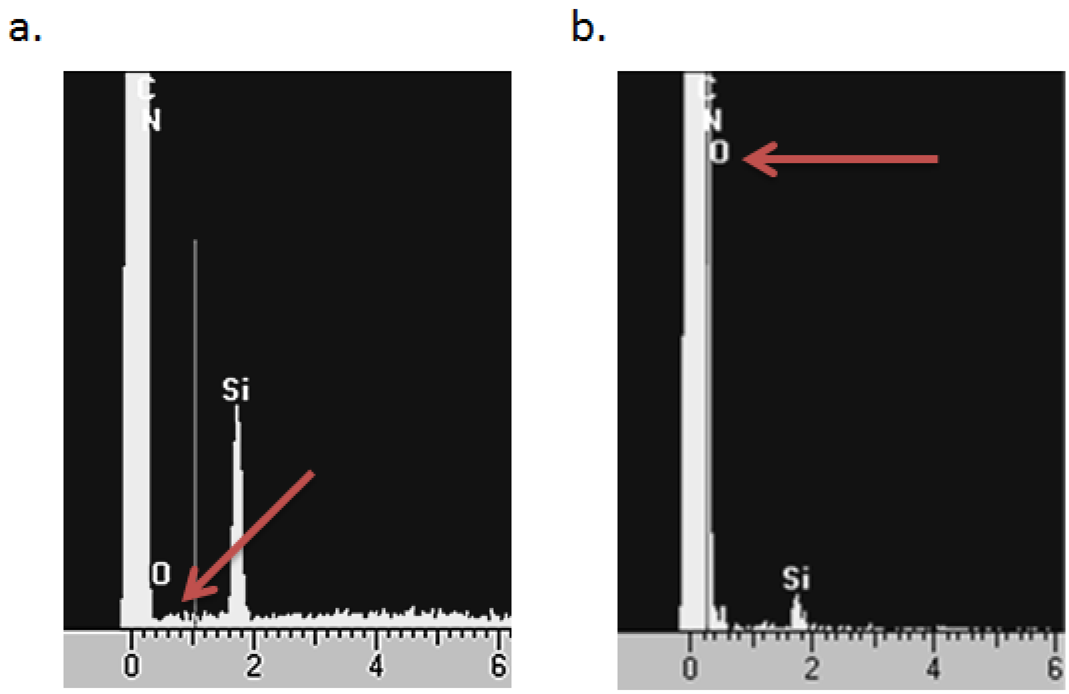

Figure S6 | EDX analysis of (**a**) CNP-PCDA, and (**b**) NCS. Red arrow identifies oxygen peak pre and post coating with PS-*b*-PAA.

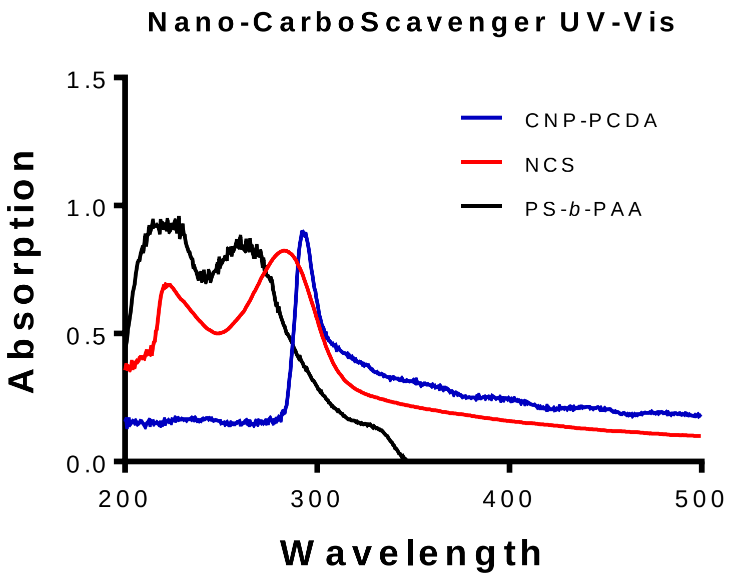

Figure S7 | An overlay UV-VIS absorption spectra of NCS and the components CNP-PCDA and PS-*b*-PAA. All measurements were taken in THF.

Zuluf/Marjan

Origin: Saudi Arabia
Synonyms: Arabian Medium

Data from OGJ 99 were originally published in 1983 as part of a series entitled "Guide to Export Crudes for the '80s".

		Data	Notes	Reference ID
**API Gravity**				
		31.1		OGJ 99
**Sulphur (weight %)**				
		2.48		OGJ 99
**Reid Vapour Pressure (kPa)**				
		58		OGJ 99
**Pour Point (°C)**				
		-29		OGJ 99
**Kinematic Viscosity (mm²/s or cSt)**				
	Temperature (°C)			
	21	19		OGJ 99
	38	11		OGJ 99
**Yield on Crude**				
	Boiling Range (°C)			
	Light naphtha (20-100)	9		OGJ 99
	Heavy naphtha (100-150)	8		OGJ 99
	Kerosene (150-235)	14		OGJ 99
	Light gas oil (235-343)	18		OGJ 99
	Heavy gas oil (343-565)	28		OGJ 99
	Residual oil (>565)	20		OGJ 99

Figure S8 | Data sheet detailing components of Saudi Arabian Medium Crude Oil.

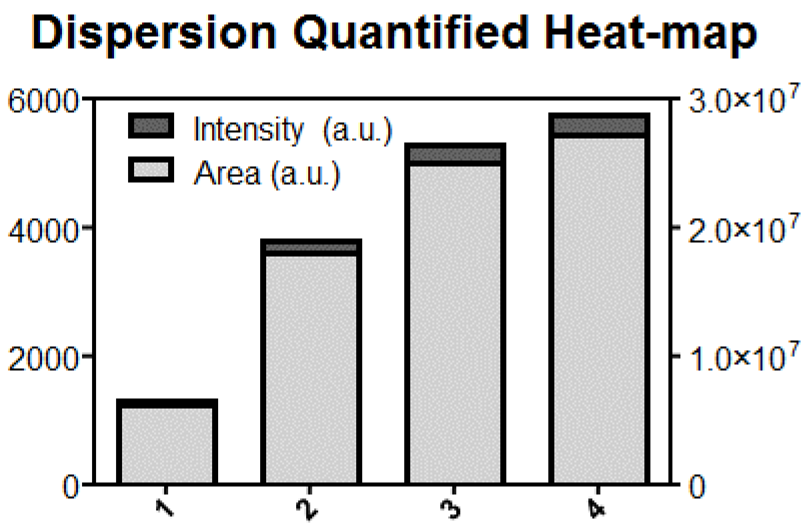

Figure S9 | Heat-map quantification of the dispersion's fluorescent images from Fig. 4d (1-4) in arbitrary units.

GENERAL CHARACTERISTICS

Specific gravity at 60°F.: 0.810
Sulphur, percent by weight: 0.14
Saybolt Universal Viscosity:
at 100°F., sec. 38 (3.46 cs)
at °F., sec.

A.P.I. gravity at 60°F.: 43.2
Pour point, °F.: 15
Colour: Brownish Green
Carbon residue, percent by weight: 0.79
(Conradson)

DISTILLATION

(U.S. Bureau of Mines Routine Method)

Stage 1 - Distillation at atmospheric pressure, 760 mm. Hg.
First drop, 33°C. (91°F.)

Fraction No.	Cut at °F.	Sum Per Cent	Specific Gravity 60/60°F.	Degrees A.P.I. °60 F.	Correlation Index	Aniline Point, °C.	Visc. S.U. 100°F.	Cloud Test, °F.	Refractive Index @ 20°C.	Dispersion $(N_F-N_C)\ 10^4$
1.	122	1.3	0.645	87.9	-	-				
2.	167	3.9	0.664	81.6	4.6	-				
3.	212	8.4	0.697	71.5	10	59.4				
4.	257	14.5	0.726	63.4	15	57.8				
5.	302	20.9	0.746	58.2	17	57.8				
6.	347	26.7	0.762	54.2	18	59.4				
7.	392	32.2	0.777	50.6	19	62.6				
8.	437	37.2	0.789	47.8	19	66.6				
9.	482	42.4	0.802	44.9	20	70.7				
10.	527	48.5	0.814	42.3	21	75.2				
Stage 2 - Distillation continued at 40 mm. Hg. pressure										
11.	392	52.5	0.829	39.2	24	78.0	38	15		
12.	437	58.1	0.837	37.6	24	82.2	43	30		
13.	482	63.4	0.850	35.0	27	86.1	51	50		
14.	527	68.0	0.860	33.0	28	89.5	67	70		
15.	572	74.0	0.871	31.0	30	93.6	106	85		
Residuum		95.8	0.922	22.0						

Carbon residue of residuum: 3.2%   Carbon residue of crude: 0.79%

APPROXIMATE SUMMARY

	Percent by Vol.	Specific Gravity	Degrees A.P.I.	Viscosity S.U. 100°F.
Light gasoline	8.4	0.679	76.9	
Total gasoline and naphtha	32.2	0.733	61.5	
Kerosine distillate	16.3	0.803	44.7	
Gas oil	11.4	0.835	38.0	
Nonviscous lubricating distillate	10.3	0.848-0.869	35-4-31.3	Below 50
Medium lubricating distillate	3.8	0.869-0.877	31.3-29.8	50-100
Viscous lubricating distillate	-	-	-	100-200
Residuum	[illegible]	[illegible]	[illegible]	Above 200

Figure S10 | Data sheet detailing components in percent by volume of Louisiana light distillate oil mixture.

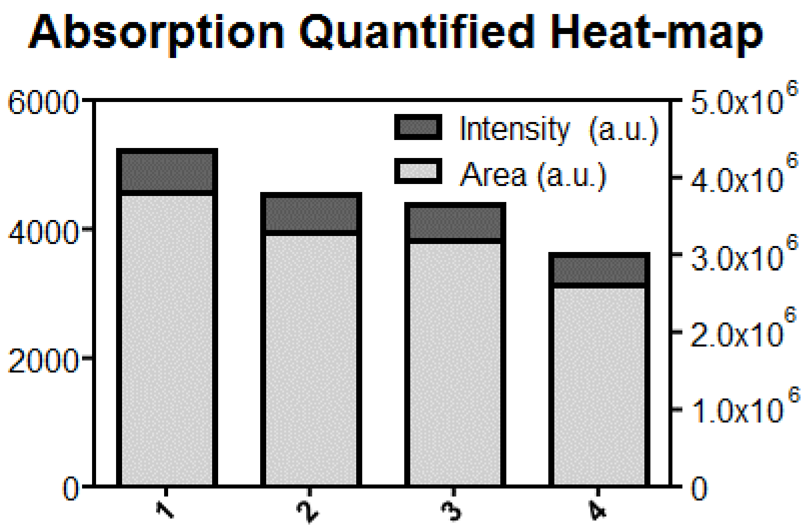

Figure S11 | Heat-map quantification of the absorption's fluorescent images from Fig. S6f (1-4) in arbitrary units.

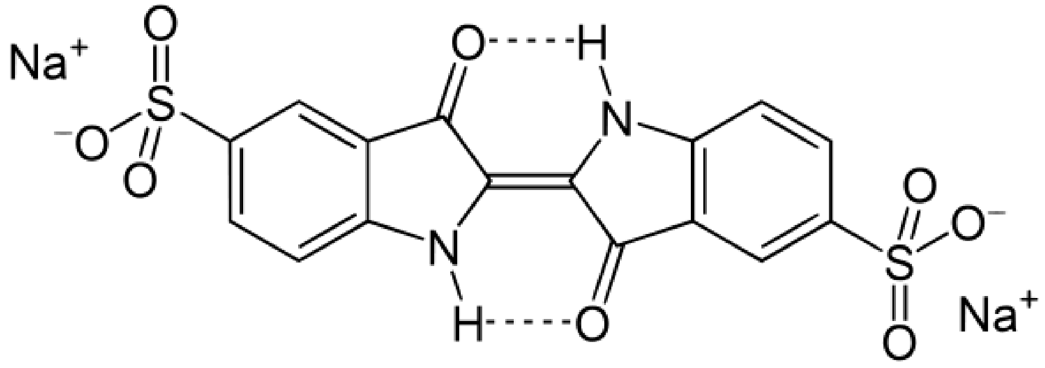

Figure S12 | Molecular structure of Indigo Carmine dye used with gasoline to visually show absorption.

Type	Agent	Remediation Ratio (Agent to Petroleum)	Toxicity (LC50)		Source
**Nanomaterial Absorption and Dispersion**	**Nano-CarboScavenger**	**1:14.75** w/w (Crude Oil) **1:9.9** w/w (Petroleum Distillate)	*V. fischeri* MCF-7 *D. rerio*	**1280 ppm** **8000 ppm** **4000 ppm**	-
Nanomaterial Absorption	Photoinduced Polymer Nanoparticle	750 mg:1 litre	*Not Calculated*	-	20
Nanomaterial Absorption	Hydrophobic Iron Core Nanoparticle	1:3.8 w/w	*Not Calculated*	-	25
Nanomaterial Absorption	Polymer - Iron Nanoparticle hybrid	1:10 w/w	*Not Calculated*	-	26
Nanomaterial Absorption	Magnetic Confined Micelle Nanoparticle	1:0.0039 w/w	*Not Calculated*	-	27
Chemical Dispersant	Accell Clean® DWD	1:10 vol/vol	*M. beryllina* *M. bahia*	8.05 ppm 1.32 ppm	65
Absorption Powder	ALSOCUP	1:10 w/w	*M. beryllina* *M. bahia*	100 ppm 100 ppm	65
Absorption Powder	OIL SOLUTIONS POWDER	1:04	*M. beryllina* *M. bahia*	22.5 ppm 2.13 ppm	65
Chemical Dispersant	BIODISPERS	1:10 vol/vol (Crude Oil) 1:20 vol/vol (Pure Solvents)	*M. beryllina* *M. bahia*	13.46 ppm 78.9 ppm	65
Absorption Powder	CAS 100©	1:10 w/w	*M. beryllina* *M. bahia*	1000 ppm 1000 ppm	65
Absorption Powder	CIAGENT	1:3 - 1:10	*M. beryllina* *M. bahia*	2227 ppm 2617 ppm	65
Chemical Dispersant	Corexit9500 EC9500A	1:10	*M. beryllina* *M. bahia*	25.20 ppm 32.23 ppm	65
Chemical Dispersant	COREXIT® EC9500B	1:10	*M. beryllina* *M. bahia*	29.13 ppm 10.00 ppm	65
Chemical Dispersant	COREXIT® EC9527A	1:10	*M. beryllina* *M. bahia*	14.57 ppm 24.14 ppm	65
Chemical Dispersant	DISPERSIT SPC 1000™	1:10	*M. beryllina* *M. bahia*	3.5 ppm 16.6 ppm	65

Chemical Dispersant	FFT-SOLUTION®	1:10	*M. beryllina*<br>*M. bahia*	5.34 ppm<br>2.72 ppm	65
Chemical Dispersant	FINASOL® OSR 52	1:05	*M. beryllina*<br>*M. bahia*	11.66 ppm<br>9.37 ppm	65
Chemical Dispersant	NEOS AB3000	125:264 vol/vol	*M. beryllina*<br>*M. bahia*	91.1 ppm<br>33.0 ppm	65
Chemical Dispersant	NOKOMIS 3-AA	5 gallons:1 acre	*M. beryllina*<br>*M. bahia*	34.22 ppm<br>20.16 ppm	65
Misc. Control Agent	PX700	1 gallon: 900 sq ft.	*M. beryllina*<br>*M. bahia*	380 ppm<br>297 ppm	65
Chemical Dispersant	SAF-RON Gold	1:10	*M. beryllina*<br>*M. bahia*	29.43 ppm<br>63.00 ppm	65
Chemical Dispersant	SEA BRAT #4	None specified	*M. beryllina*<br>*M. bahia*	30.00 ppm<br>14.00 ppm	65
Absorption Powder	WASTE-SET #3200®	1:05	*M. beryllina*<br>*M. bahia*	10000 ppm<br>5431 ppm	65

Table S2 | Table comparison of nanomaterials, commercial powder absorbents, and commercial chemical dispersants with remediation ratio (agent to petroleum) and reported toxicity. Remediation ratio is reported as weight per weight (w/w) or volume to volume (v/v) ratios. Toxicity for *M. beryllina and M. bahia is reported as their respective* LC_{50} *values.*
